# ^68^Ga-PSMA PET/CT-based multivariate model for highly accurate and noninvasive diagnosis of clinically significant prostate cancer in the PSA gray zone

**DOI:** 10.1186/s40644-023-00562-x

**Published:** 2023-09-04

**Authors:** Jinhui Yang, Jian Li, Ling Xiao, Ming Zhou, Zhihui Fang, Yi Cai, Yongxiang Tang, Shuo Hu

**Affiliations:** 1 Department of Nuclear Medicine, Xiangya Hospital, Central South University, Changsha, Hunan China; 2 Department of Urology, Xiangya Hospital, Central South University, 87 Xiangya Road, Changsha, 410008 Hunan China; 3 Key Laboratory of Biological Nanotechnology of National Health Commission, Xiangya Hospital, Central South University, Changsha, Hunan China; 4 National Clinical Research Center for Geriatric Disorders (XIANGYA), Xiangya Hospital, Central South University, Changsha, Hunan China; 5 Department of Nuclear Medicine (PET Center), Key Laboratory of Biological Nanotechnology of National Health Commission, XiangYa Hospital, Central South University, 87 Xiangya Road, Changsha, 410008 Hunan China

**Keywords:** Prostate cancer, Clinically significant prostate cancer, PSMA, PET/CT, Gray zone

## Abstract

**Background:**

The prostate-specific antigen (PSA) has been widely used in screening and early diagnosis of prostate cancer (PCa). However, in the PSA grey zone of 4–10 ng/ml, the sensitivity and specificity for diagnosing PCa are limited, resulting in considerable number of unnecessary and invasive prostate biopsies, which may lead to potential overdiagnosis and overtreatment. We aimed to predict clinically significant PCa (CSPCa) by combining the maximal standardized uptake value (SUVmax) based on ^68^Ga‑PSMA PET/CT and clinical indicators in men with gray zone PSA levels.

**Methods:**

81 patients with suspected PCa based on increased serum total PSA (TPSA) levels of 4 − 10 ng/mL who underwent transrectal ultrasound/magnetic resonance imaging (MRI)/PET fusion-guided biopsy were enrolled. Among them, patients confirmed by histopathology were divided into the CSPCa group and the non-CSPCa group, and data on PSA concentration, prostate volume (PV), PSA density (PSAD), free PSA (FPSA)/TPSA, Prostate Imaging-Reporting and Data System version 2.1 (PI-RADS v2.1) score, ^68^Ga-PSMA PET/CT imaging evaluation results and SUVmax were compared. Multivariate logistic regression analysis was performed to identify the independent predictors for CSPCa, thereby establishing a predictive model based on SUVmax that was evaluated by analyzing the receiver operating characteristic (ROC) curve and decision curve analysis.

**Results:**

Compared to non-CSPCa, CSPCa patients had smaller PVs (median, 31.40 mL), lower FPSA/TPSA (median, 0.12), larger PSADs (median, 0.21 ng/mL^2^) and higher PI-RADS scores (P < 0.05). The prediction model comprising ^68^Ga-PSMA PET/CT maximal standardized uptake value, PV and FPSA/TPSA had the highest AUC of 0.927 compared with that of other predictors alone (AUCs of 0.585 for PSA, 0.652 for mpMRI and 0.850 for 68Ga-PSMA PET/CT). The diagnostic sensitivity and specificity of the prediction model were 86.21% and 86.54%, respectively.

**Conclusion:**

Given the low diagnostic accuracy of regular PSA tests, a new prediction model based on the ^68^Ga-PSMA PET/CT SUVmax, PV and FPSA/TPSA was developed and validated, and this model could provide a more satisfactory predictive accuracy for CSPCa. This study provides a noninvasive prediction model with high accuracy for the diagnosis of CSPCa in the PSA gray zone, thus may be better avoiding unnecessary biopsy procedures.

**Supplementary Information:**

The online version contains supplementary material available at 10.1186/s40644-023-00562-x.

## Introduction

Prostate cancer (PCa) is the second most common cancer and the fifth leading cause of cancer death among males around the world [1]. Serum prostate-specific antigen (PSA) and digital rectal examination have been widely used in PCa screening and as classical parameters for the early diagnosis of PCa. This has led to a dramatic decrease in the number of PCa-related deaths. However, the PSA concentration increases not only in PCa but also in some nonmalignant conditions, such as benign prostatic hyperplasia (BPH), prostatic hyperplasia, urinary tract infection, and indwelling catheters, especially among patients with PSA levels within the gray zone of 4 − 10 ng/mL [2]. The low specificity of the PSA test can lead to unnecessary and invasive biopsies, which may cause pain, bleeding, infection and potential tumor seeding. In addition, previous studies have revealed that fewer than 30% of males with PSA levels in the gray zone have pathologically confirmed PCa, and the detection rate for clinically significant prostate cancer (CSPCa) is much lower [3]. Therefore, the low specificity and sensitivity of the PSA test can lead to potential overdiagnosis and overtreatment, and there is an urgent need for a highly accurate and noninvasive alternative for the early diagnosis of PCa and CSPCa and to reduce unnecessary biopsies, especially for patients who fall within the PSA gray zone.

Multiparametric magnetic resonance imaging (mpMRI) is currently the optimum imaging technology for the diagnosis and monitoring of PCa [4, 5]. Studies have shown that mpMRI sensitivity for the detection of PCa is up to 96%, while the specificity is only 36–58%, indicating a relatively high false-positive rate [6, 7]. MRI-based prediction models combining clinical parameters have been developed and shown good predictive ability for patients with PSA levels within the gray zone [8, 9]. However, regarding diagnostic accuracy, the usefulness of these models is controversial [10, 11], and the prostate biopsy for confirming PCa still tends to rely on PSA-based specificity [12, 13].

Gallium-68 prostate-specific membrane antigen (^68^Ga-PSMA) positron emission tomography/computed tomography (PET/CT) is currently revolutionizing the PCa diagnostic pathway and has shown value as a means of detecting biochemical recurrence and staging of high-risk PCa [14, 15], and it is the most promising method for identifying patients with CSPCa among suspected PCa patients with a negative mpMRI [16]. However, studies have reported the limitations of ^68^Ga-PSMA PET/CT in detecting low- and/or intermediate-risk PCa [17], which greatly decreases its usefulness as a first-line PCa diagnostic tool. We hypothesized that incorporating additional clinical predictive indicators would improve the performance of the ^68^Ga-PSMA PET/CT-based multivariate model for the diagnosis of PCa within the PSA gray zone, especially CSPCa.

Herein, we focus on the so-called “PSA gray zone” to develop a predictive model combining ^68^Ga-PSMA PET/CT with traditional clinical risk factors, aiding in the highly sensitive, specific and noninvasive diagnosis of CSPCa within the PSA gray zone to reduce the number of unnecessary prostate biopsies.

## Materials and methods

### Subjects

We performed a review of consecutive patients with two or more tests showing increased serum total PSA (TPSA) levels between 4 and 10 ng/mL from September 2019 to January 2022. All enrolled patients underwent prostate mpMRI, ^68^Ga-PSMA PET/CT and systematic transrectal ultrasound (TRUS)/MRI/PET fusion-guided prostate biopsy before a pathological diagnosis was finally obtained. Patients for whom the biopsy results were negative have undergone a minimum of 1-year follow-up of PSA and mpMRI. The exclusion criteria were as follows (a) patients who had prior treatment before ^68^Ga-PSMA PET/CT, mpMRI, and biopsy, such as radiotherapy, chemotherapy, or androgen deprivation therapy; (b) an interval time among the ^68^Ga-PSMA PET/CT, mpMRI and biopsy procedures exceeding three months; (c) patients who had missing clinical data or nonstandard examinations. The study protocol was approved by the Ethics Committee of Xiangya Hospital, and all patients provided written informed consent.

### mpMRI examination and image evaluation

All patients underwent prostate mpMRI on a 3.0-T MRI scanner (Siemens Healthineers, GE) by using an external coil. Two experienced radiologists who were blinded to the patient’s clinical data and pathological results independently reported the Prostate Imaging-Reporting and Data System (PI-RADS) score as described in the version 2.1 protocol. In cases of disagreement, the final PI-RADS score was determined through joint assessment and consensus. Lesions with a score of 1 − 2 were considered negative, while lesions with a score of 3 − 5 were considered positive in mpMRI. Prolate ellipsoid formula (length×width×height×0.52) was used to assess the prostate volume (PV) on T2-weighted images. PSA density (PSAD) was defined as the ratio of PSA to PV.

### ^68^Ga-PSMA PET/CT examination and image evaluation

All ^68^Ga-PSMA PET/CT scans were performed before the prostate biopsy. A ^68^Ga-PSMA-617 of 3.7 − 4.44 MBq/kg was administered to each patient. Scans were performed sequentially 40 ± 10 min later using a PET/CT scanner (General Electric Healthcare, 690 Elite, Waukesha, WI, USA). The detailed mpMRI and PET/CT protocols were described in our previous study [18].

PET/CT images were independently evaluated by two nuclear medicine physicians who were blinded to the pathology and other clinical findings. The negative lesions were defined as follows (a) no dominant intraprostatic activity; (b) diffuse transition zone activity or symmetrical central zone activity that does not extend to the prostate margin on CT. The positive lesions were defined as follows (a) focal transition zone activity visually twice above background; (b) focal peripheral zone activity (no minimum intensity); (c) intense uptake (visual very high intensity or maximal standardized uptake value [SUVmax] > 12) [19]. The SUVmax of all suspicious lesions and the prostate gland background for negative patients were measured. The dominant lesion SUVmax was recorded for further analysis.

### Histopathology examination

After undergoing MRI/PET examination, a urologist who had performed over 1000 transperineal prostate systematic biopsies (SBs) and 300 targeted biopsies (TBs) performed transperineal prostate SBs with 12 cores. Additional PET and MRI TB were performed for positive patients, and each positive lesion included 2 − 4 cores. All biopsies were performed using a BK Fusion Biopsy system. All patients with biopsy-proven CSPCa in this study subsequently received radical prostatectomy, and data on the Gleason score and International Society of Urological Pathology grade group of prostatectomy specimens were collected for further analysis [20]. CSPCa was defined as Gleason group > 1, while cases of prostatitis, BPH, or PCa classified into the Gleason group = 1 were considered non-CSPCa.

### Statistical analysis

T tests/Spearman rank tests and Chi-square/Fisher’s exact tests were used to compare the clinical and imaging features between two groups. Kappa analysis of the consistency of PI-RADS score and ^68^Ga-PSMA PET/CT results was made. Multivariate binary logistic regression analysis was performed to determine independent predictors, and then an SUVmax-based prediction model was established. The areas under the receiver operating characteristic (ROC) curve (AUC) were used to assess the diagnostic value for CSPCa. Decision curve analysis (DCA) was performed to assess the clinical utility of each diagnostic method. A p value < 0.05 was considered statistically significant.

## Results

### Participant characteristics and stratification

A total of 81 men were finally enrolled in this study. The clinical and imaging characteristics of all patients are presented in Table 1. Twenty-nine (35.8%) men showed CSPCa on prostate pathology, and 52 (64.2%) men showed non-CSPCa, including 8 (9.9%) with low-grade PCa (Gleason score 3 + 3) and 44 (54.3%) with BPH or acute or chronic prostatitis. The kappa values were 0.765 (P < 0.001) in PI-RADS score, and 0.876 (P < 0.001) in ^68^Ga-PSMA PET/CT results, respectively, suggesting good inter-reader agreements.

**Table 1:** Patient demographic and clinical information

Characteristics	Value
*Clinical data*	
**Mean age (SD), y**	63.91 (6.45)
**Median PSA (IQR), ng/ml**	6.92 (5.29–8.28)
**Median PV (IQR), ml**	39.23 (30.03–61.36)
**Median PSAD (IQR), ng/ml** ^**2**^	0.15 (0.11–0.25)
**Median FPSA/TPSA (IQR)**	0.15 (0.12–0.20)
*Imaging characteristics*	
**PI-RADS 1–2**	27 (33.3%)
**PI-RADS 3**	23 (28.4%)
**PI-RADS 4**	16 (19.8%)
**PI-RADS 5**	15 (18.5%)
**SUVmax**	5.5 (3.5–8.3)
*Pathological characteristics*	
**Gleason score**	
**0**	44 (54.3%)
**6**	8 (9.9%)
**7**	22 (27.2%)
**8**	4 (4.9%)
**9**	3 (3.7%)
**10**	0 (0)
**Gleason group**	
**0**	44 (54.3%)
**1**	8 (9.9%)
**2**	17 (21.0%)
**3**	2 (2.5%)
**4**	4 (4.9%)
**5**	3 (3.7%)

SD, standard deviation; PSA, prostate-specific antigen; IQR, interquartile range; PV, prostate volume; PSAD, prostate-specific antigen density; TPSA, total prostate-specific antigen; FPSA, free prostate-specific antigen; mpMRI, multiparametric magnetic resonance imaging; PI-RADS, Prostate Imaging-Reporting and Data System; SUVmax, maximum standardized uptake values

### Multivariate regression analysis and prediction model establishment

The results of the clinical indicators, PI-RADS score and ^68^Ga-PSMA PET/CT evaluation in the comparison of the CSPCa and non-CSPCa groups are shown in Table 2**and** Table 3. These results show that PSA level and age were comparable between two groups. This is why new accurate approaches and biomarkers are needed. The PSAD, PV, PI-RADS scores, ^68^Ga-PSMA PET/CT results and SUVmax were significantly different between the two groups (P < 0.05).

**Table 2:** Characteristics according to pathologic reports in patients with gray zone PSA levels

	Non-CSPCa	CSPCa	P value
**Mean age (SD), y**	63.23 (6.71)	65.14 (5.87)	0.204
**Median PSA (IQR), ng/ml**	6.29 (5.24–7.95)	7.61 (5.34–9.21)	0.206
**Median PV (IQR), ml**	49.66 (32.90-68.05)	31.40 (21.69–37.32)	< 0.001*
**Median PSAD (IQR), ng/ml** ^**2**^	0.13 (0.09–0.17)	0.21 (0.17–0.29)	< 0.001*
**Median FPSA/TPSA (IQR)**	0.16 (0.13–0.22)	0.12 (0.09–0.15)	0.001*
*MRI results*			0.005*
**PI-RADS 1–2**	23 (44.2%)	4 (13.8%)	
**PI-RADS 3–5**	29 (55.8%)	25 (86.21%)	
^*68*^ *Ga-PSMA PET/CT results*			< 0.001*
**Negative**	40 (76.92%)	2 (6.90%)	
**Positive**	12 (23.08%)	27 (93.10%)	
**SUVmax**	3.75 (3.20–6.20)	8.20 (6.50–12.70)	< 0.001*

CSPCa, clinically significant prostate cancer; SD, standard deviation; PSA, prostate-specific antigen; IQR, interquartile range; PV, prostate volume; PSAD, prostate-specific antigen density; TPSA, total prostate-specific antigen; FPSA, free prostate-specific antigen; MRI, magnetic resonance imaging; PI-RADS, Prostate Imaging-Reporting and Data System; ^68^Ga-PSMA PET/CT, ^68^Ga-labeled prostate-specific membrane antigen positron emission tomography/computed tomography; SUVmax, maximum standardized uptake values

* p < 0.05

**Table 3:** Logistic regression analysis for predicting CSPCa

	Univariate analysis					Multivariate analysis	
	OR (95% CI)	P value				OR (95% CI)	P value
**Age (years)**	1.05 (0.98–1.13)		0.204			-	-
**PSA**	1.17 (0.92–1.49)		0.209			-	-
**PV (ml)**	0.95 (0.92–0.98)		0.002*			0.95 (0.91-1.00)	0.032*
**PSAD (ng/ml** ^**2**^ **)**						-	-
**<0.15**	reference		-			-	-
**≥0.15**	12.71 (3.31–48.76)		< 0.001*			-	-
**FPSA/TPSA**	0.00 (0.00-0.03)		0.005*			0.00 (0.00-0.05)	0.016*
**SUVmax**	1.50 (1.23–1.83)		< 0.001*			1.73 (1.33–2.25)	< 0.001*

CSPCa, clinically significant prostate cancer; PSA, prostate-specific antigen; PV, prostate volume; PSAD, prostate-specific antigen density; TPSA, total prostate-specific antigen; FPSA, free prostate-specific antigen; SUVmax, maximum standardized uptake values

* p < 0.05

The univariate analysis revealed that PV, free prostate-specific antigen (FPSA)/TPSA, high PSAD (≥ 0.15ng/ml^2^) and SUVmax (all P < 0.05) were risk factors for CSPCa. Subsequently, the significant variables from the univariate analysis (PV, FPSA/TPSA, and SUVmax) were included in the multivariate logistic regression models. The changes in PSAD were influenced both by the PV and TPSA, and PSAD was not included in the multivariate analysis. As shown in Table 3, the independent predictors for CSPCa were PV (odds ratio [OR]: 0.95, 95% confidence interval [CI]: 0.91-1.00), FPSA/TPSA (OR: 0.00, 95%CI: 0.00-0.05) and SUVmax (OR: 1.73, 95%CI: 1.33–2.25) (P < 0.05). Then, a ^68^Ga-PSMA PET/CT SUVmax-based prediction model was established to predict CSPCa.

### Diagnostic performance of the predictive model

**Fig. 1:**
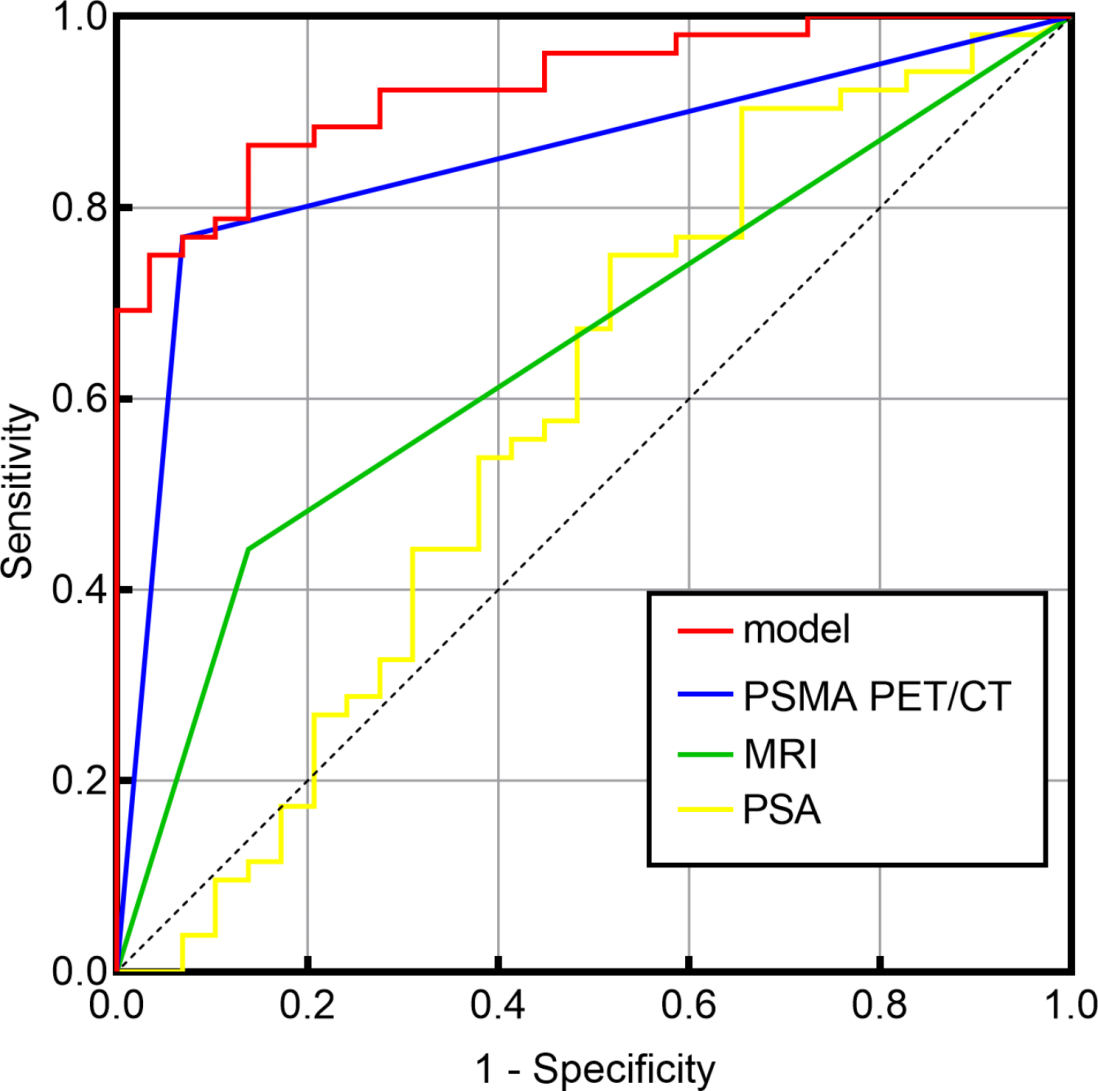
ROC of prediction model and each indicator for detecting CSPCa in patients with PSA grey zone. The prediction model is the combination of the ^68^Ga-PSMA PET/CT SUVmax, PV and FPSA/TPSA. ROC, receiver operating characteristic; ^68^Ga-PSMA, ^68^Ga-labeled prostate-specific membrane antigen; PET/CT, positron emission tomography/computed tomography; MRI, magnetic resonance imaging; CSPCa, clinically significant prostate cancer; PSA, prostate-specific antigen

Analysis of ROC curves showed that the AUC for PSA was 0.585 (95%CI: 0.447–0.724) (Table 4). More powerful univariate independent predictor associated with CSPCa risk was ^68^Ga-PSMA PET/CT (AUC = 0.850), which was higher than that of PSA and mpMRI (AUC = 0.652). When the ^68^Ga-PSMA PET/CT SUVmax was combined with PV and FPSA/TPSA, the AUC (0.927) of the prediction model was the highest compared with the univariate independent predictors (Fig. 1).

**Table 4:** Diagnostic performance of the prediction model in diagnosing CSPCa in PSA gray zone patients

	Sensitivity (95% CI)	Specificity (95% CI)	AUC (95% CI)
**Model**	86.21% (69.44-94.50%)	86.54% (74.73-93.32%)	0.927 (0.8738-0.980)
^**68**^ **Ga-PSMA PET/CT**	93.10% (76.97-99.15%)	76.92% (63.16-87.47%)	0.850 (0.762–0.938)
**mpMRI**	86.21% (68.34-96.11%)	44.23% (31.59-57.67%)	0.652 (0.532–0.773)
**PSA**	48.28% (31.39-65.57%)	75.00% (61.79-84.77%)	0.585 (0.447–0.724)

CSPCa, clinically significant prostate cancer; PSA, prostate-specific antigen; ^68^Ga-PSMA PET/CT, ^68^Ga-labeled prostate-specific membrane antigen positron emission tomography/computed tomography; mpMRI, multiparametric magnetic resonance imaging

**Fig. 2:**
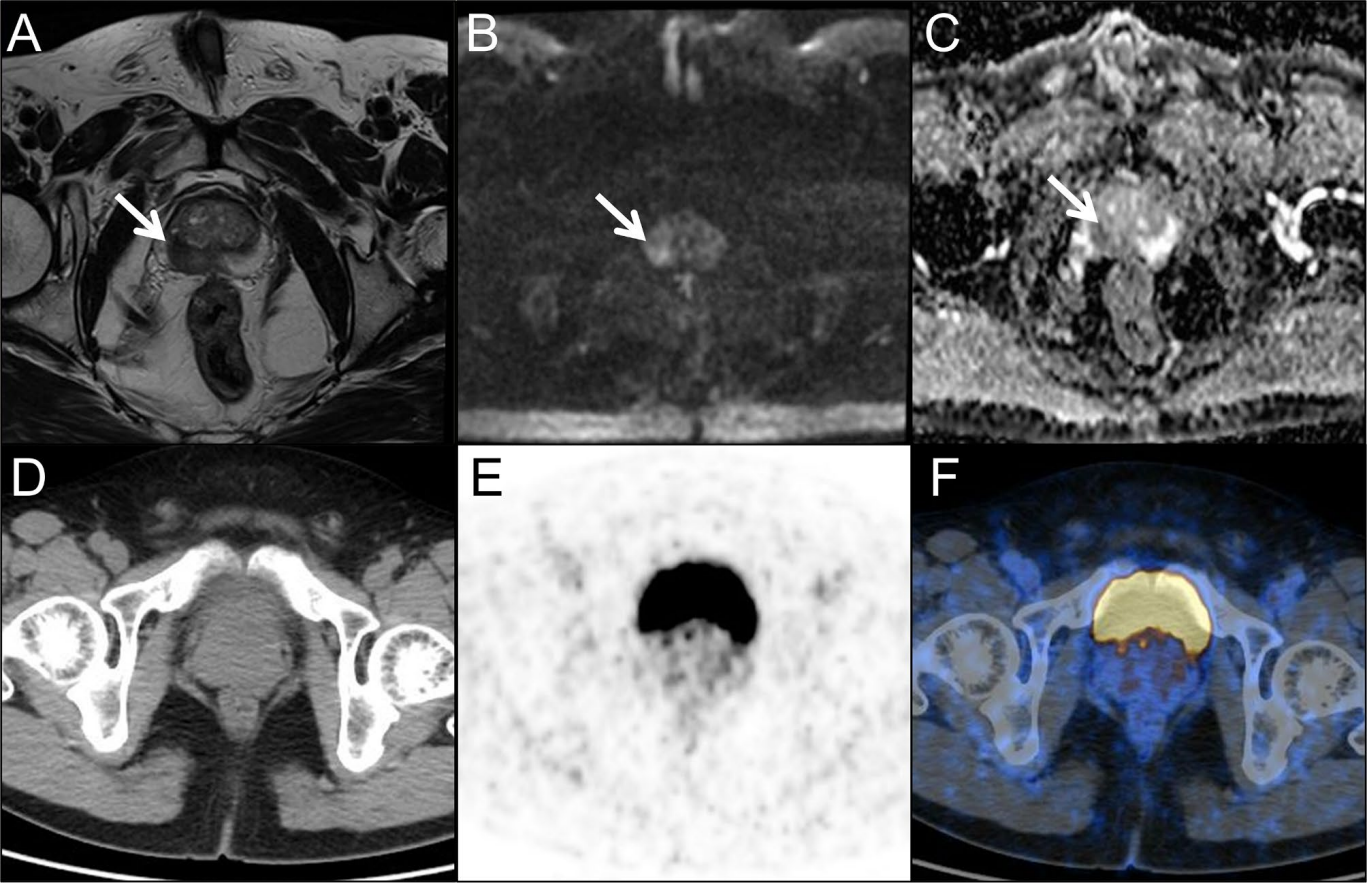
A 66-year-old man presented with a persistently elevated prostate-specific antigen (PSA) range of 7.08–8.58 ng/ml over a period of six months. T2-weighted imaging (A) revealed a hypointense lesion in the right periphery of the prostate (arrow) showing hyperintense on the diffusion weighted imaging (DWI) (B, arrow), and hypointense on the apparent diffusion coefficient (ADC) maps (C, arrow) resulting in a Prostate Imaging-Reporting and Data System (PI-RADS) score of 4. However, positron emission tomography/computed tomography (PET/CT) images (D, CT; E, PET; F, fusion) showed no prostate-specific membrane antigen (PSMA) uptake in the involved region. According to the predictive model, the probability of clinically significant prostate cancer for this lesion is 0.10 (< 0.35). Subsequent prostate biopsy result in benign prostatic hyperplasia accompanied by prostatitis. Following 1-year of symptomatic treatment, the patient’s PSA returned to normal

**Fig. 3:**
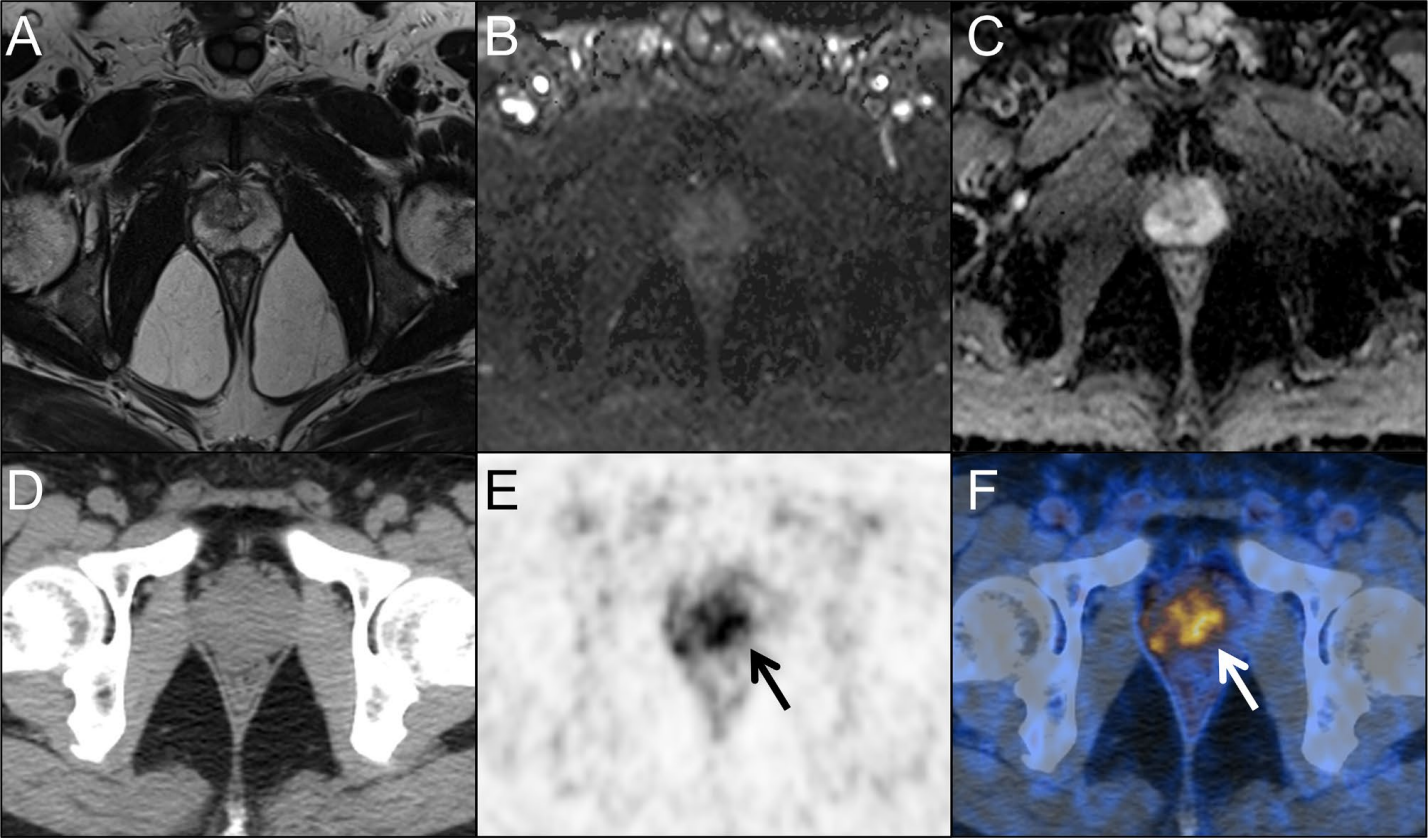
A 55-year-old man presented with a persistently elevated prostate-specific (PSA) range of 4.37–6.82 ng/ml over a period of one year. Magnetic resonance imaging (MRI) (A, T2-weight imaging; B, DWI imaging; C, ADC map) didn’t revealed the presence of any malignant lesions. Positron emission tomography/computed tomography (PET/CT) images (D, CT; E, PET; F, fusion) showed intensive prostate-specific membrane antigen (PSMA) uptake in the center gland and right periphery of the prostate (arrows). According to the predictive model, the probability of clinically significant prostate cancer for this lesion is 0.67 (> 0.35). Subsequent prostate biopsy result in a Gleason score 4 + 3 prostate cancer

These univariate independent indicators and ^68^Ga-PSMA PET/CT SUVmax-based prediction models were applied to evaluate their diagnostic performances for CSPCa patients with PSA values in the gray zone (Table 4; Fig. 1). When the cutoff value of PSA was divided by the optimal threshold of 7.77 ng/mL of the Youden index, it had 48.28% sensitivity and 75.00% specificity for diagnosing CSPCa, whereas PI-RADS scores ≥ 3 had values of 86.21% and 44.23%, respectively. Table 4 shows that the Visual evaluation of ^68^Ga-PSMA PET/CT had the highest sensitivity (93.10%) compared with that of the independent indicators and a moderate specificity of 76.92%. In addition, we also performed a semiquantitative analyses of ^68^Ga-PSMA PET/CT. At the cuttoff value of SUVmax 5.15, the sensitivity and specificity of ^68^Ga-PSMA PET/CT for diagnosing CSPCa were 93.10% and 73.10%, respectively. Furthermore, the ^68^Ga-PSMA PET/CT SUVmax-based prediction model (combination SUVmax with PV and FPSA/TPSA) had 86.21% sensitivity and 86.54% specificity for diagnosing CSPCa at a cutoff value of 0.35. During the early diagnosis of CSPCa, the ^68^Ga-PSMA PET/CT SUVmax-based prediction model is a much better diagnostic method than PSA-based tests, as revealed by the larger AUC value and higher sensitivity and specificity of this test than those of PSA- and mpMRI-based tests. Figures 2 and 3 present two typical cases of the study.

**Fig. 4:**
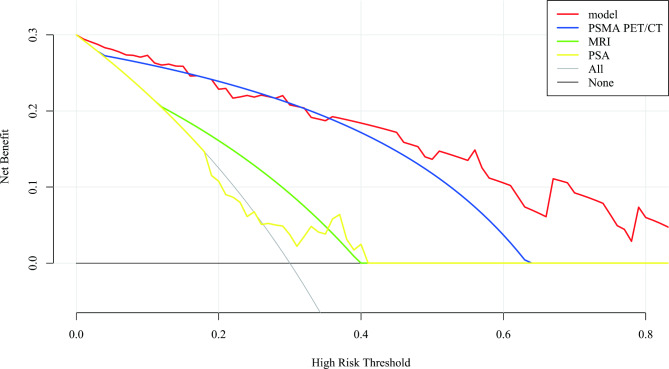
DCA of prediction model and each indicator for detecting CSPCa in patients with PSA grey zone. The prediction model is the combination of the ^68^Ga-PSMA PET/CT SUVmax, PV and FPSA/TPSA. DCA, decision curve analysis; ^68^Ga-PSMA, ^68^Ga-labeled prostate-specific membrane antigen; PET/CT, positron emission tomography/computed tomography; MRI, magnetic resonance imaging; CSPCa, clinically significant prostate cancer; PSA, prostate-specific antigen

As shown in Fig. 4, DCA was performed to compare the clinical utility of PSA, MRI, ^68^Ga-PSMA PET/CT and SUVmax-based prediction models in assisting prostate biopsy decisions at PSA values in the gray zone. The ^68^Ga-PSMA PET/CT and prediction models had comparable and obviously higher net benefits than the other diagnostic methods for risk thresholds of 10-40%. For risk thresholds greater than or equal to 40%, the net benefit of the prediction model was greater than that of ^68^Ga-PSMA PET/CT and the other diagnostic methods.

## Discussion

PCa requires a multidisciplinary approach, in which many specialties play a key role [21]. In the present study, we have developed a simple predictive model combining the SUVmax based on ^68^Ga-PSMA PET/CT with traditional clinical risk factors, which can achieve a highly accurate and noninvasive diagnosis of CSPCa in patients falling within the PSA gray zone. This model can meet clinical demands by taking advantage of ^68^Ga-PSMA PET/CT, with high specificity and sensitivity. PSA test results in a highly negative biopsy rate, but the combination of ^68^Ga-PSMA PET/CT and PSA tests provides a good performance-efficiency-diagnostic model to improve the net benefit and reduce the number of unnecessary prostate biopsies.

PSA alone in the gray zone is not appropriate for diagnosing CSPCa. Our AUC for the PSA value was 0.585 when the cutoff value of PSA was divided by the optimal threshold of 7.77 ng/mL, which had low sensitivity and specificity for diagnosing CSPCa. The results were consistent with previous reports [22, 23]. And other clinical indicators have been proven to offer better guidance in the diagnosis of CSPCa. Some researchers have clarified that decreased PV was associated with a higher risk of CSPCa [24, 25]. The outcomes based on a larger PV corresponding to a lower risk of CSPCa were a result of a higher proportion of low-volume cancers in larger prostates. However, C.-g. Wei et al. showed that PSAD and FPSA/TPSA were not effective for CSPCa with PSA levels in the gray zone [26]. PSAD is related to both PSA and PV. When the TPSA value is small, the relationship between PSAD and PV is closer; that is, when the TPSA value is 4 − 10 ng/mL, the changes in PSAD are more influenced by the PV. In addition, differences in sample selection and the effects of race may also be responsible for the opposite results. Hence, the diagnostic value of these clinical indicators for predicting PCa or CSPCa risk remains controversial and unsatisfactory in many studies, especially in TPSA levels within the “gray zone”.

Considering the well-documented limitations of clinical indicators in the diagnosis of CSPCa among cases with PSA gray zones, there is a concerted effort to develop alternative diagnostic tools [4]. Studies have reported that mpMRI can improve the sensitivity (93%) and positive predictive values of diagnosing PCa, especially for CSPCa [27]. However, the diagnostic performance of mpMRI for assessing CSPCa remains debatable, owing to its diagnostic subjectivity and low specificity [28]. Our results also showed that the sensitivity of mpMRI for the diagnosis of CSPCa was up to 86.21% when the PI-RADS score was selected as 3 or higher, while the specificity was only 44.23%. This could be because the imaging effect of mpMRI is dependent on the combination of contrast agents and the density of water molecules in the body. Some benign lesions, such as prostatitis, postprostate puncture bleeding, and prostatic hyperplasia [29], can cause signal changes similar to those of malignant tumors, accounting for the majority of people with PSA levels in the gray zone. Meanwhile, it must be acknowledged that the PI-RADS score also depends on the experience and diagnostic ability of the radiologists.

As an imaging biomarker, ^68^Ga-PSMA PET/CT has been used to differentiate malignant from benign lesions. Since PSMA expression is much higher in most PCa cells than in normal prostate tissue and benign prostate lesions [30], PSMA-targeted PET has high specificity for both PCa and CSPCa [31, 32]. These findings suggested that ^68^Ga-PSMA PET/CT may have a better ability and stability than mpMRI to identify CSPCa in patients with PSA gray zone (specificity: 76.92% vs. 44.23%), avoiding more false-positive instances and reducing overdiagnosis.

In challenging tumors, such as PCa, there is the need for new powerful biomarkers to be able to improve patient management. Combining imaging, analytical and clinical parameters can provide new biomarkers [33, 34]. In this study, we have established a predictive model that combines imaging and clinical parameters. Multivariate models have exhibited the unique capability of data analysis to improve the clinical diagnosis of diseases compared to a single indicator variable [8, 9]. As shown in Fig. 1, our study found that combining the SUVmax based on ^68^Ga-PSMA PET/CT, PV and FPSA/TPSA could improve the diagnosis of CSPCa (AUC = 0.927), which is significantly better than the predictive power of either ^68^Ga-PSMA PET/CT (AUC = 0.850) or mpMRI alone (AUC = 0.652). Although the sensitivity (93.10%) of ^68^Ga-PSMA PET/CT image evaluation exceeds that of the model (86.21%), in clinical practice, visual evaluation is performed first, and then the ^68^Ga-PSMA PET/CT-based multivariate model is established. This combined analysis has important clinical significance for reducing unnecessary prostate biopsies because of the improvement in specificity. Various clinical factors were combined with SUVmax to better identify CSPCa and guide clinical decisions in patients with gray areas of PSA, which may avoid unnecessary invasive procedures.

There are some limitations to this study. First, the retrospective single-center study and relatively small sample size limited our statistical validation to some extent. Second, the patients included in this study cohort were not chosen completely at random, and the positive CSPCa puncture rate of patients in this study was significantly higher than that of other studies, which may lead to bias in indicators such as positive predictive values and negative predictive values. Third, we used multiple biopsy methods as pathological reference standards [18], which may overlook some patients with CSPCa compared to prostatectomy specimens.

## Conclusions

In conclusion, a new prediction model based on ^68^Ga-PSMA PET/CT SUVmax, PV and FPSA/TPSA was developed and validated, and it can provide a more satisfactory predictive accuracy for CSPCa in men with PSA levels within the gray zone, thus better avoiding unnecessary biopsy procedures.

### Electronic supplementary material

Below is the link to the electronic supplementary material.

Supplementary Material 1

## Data Availability

The datasets generated during and/or analyzed during the current study are available from the corresponding author on reasonable request.

## Abbreviations

PCa: Prostate cancer
PSA: Postate-specific antigen
BPH: Benign prostatic hyperplasia
CSPCa: Clinically significant prostate cancer
mpMRI: Multiparametric magnetic resonance imaging
^68^Ga-PSMA: Gallium-68 prostate-specific membrane antigen
PET/CT: Positron emission tomography/computed tomography
TPSA: Total PSA
TRUS: Transrectal ultrasound
PI-RADS: Prostate Imaging-Reporting and Data System
PV: Prostate volume
PSAD: PSA density
SUVmax: Maximal standardized uptake value
SBs: Systematic biopsies
TBs: Targeted biopsies
ROC: Receiver operating characteristic
AUC: Areas under the curve
DCA: Decision curve analysis
FPSA: Free prostate-specific antigen
OR: Odds ratio
