# Supplementary material for: 68Ga-PSMA PET/CT-based multivariate model for highly accurate and noninvasive diagnosis of clinically significant prostate cancer in the PSA gray zone
Source: Cancer Imaging. 2023 Sep 4;23:81. doi: 10.1186/s40644-023-00562-x (PMC10476329; doi:10.1186/s40644-023-00562-x)
Supplement: Supplementary file 1 — Supplementary Material 1 [file 40644_2023_562_MOESM1_ESM.docx]

**Dear Prof. Rodney Hicks and Prof. Annick Van den Abbeele:**

**RE: No. CAIG-D-23-00019**

We would like to thank you and the expert reviewers for the examination and approval of our manuscript entitled, “^68^Ga-PSMA PET/CT-based multivariate model for highly accurate and noninvasive diagnosis of clinically significant prostate cancer in the PSA gray zone” by Yang et al. We built a new prediction model based on ^68^Ga-PSMA PET/CT SUVmax, PV and FPSA/TPSA, which provide a more satisfactory predictive accuracy for CSPCa in men with PSA levels within the gray zone, thus better avoiding unnecessary biopsy procedures.

We thank the reviewers and editors for the critical reading of our paper and for the very valuable suggestions we received. Based on the helpful comments, we have carefully revised the manuscript and provided a point-by-point response to the reviewers' comments. Dr Jian Li did a great and distinct contribution to the new version after major revision. After consultations, all authors agreed with the rearrangement of the names. In the final version of the article, Jian Li is tagged as co-first author. We herewith submit the revised manuscript for reexamination. We believe that these revisions have significantly strengthened the manuscript, and hope you find it acceptable for publication in *Cancer Imaging*.

We are grateful to you for allowing us to revise our manuscript and look forward to hearing from you soon.

Sincerely

Yi Cai, Yongxiang Tang and Shuo Hu

We thank the reviewers for their thorough examination of our manuscript, and the valuable and insightful comments provided. We have revised the manuscript accordingly and provide a point-to-point response to the comments below.

**Reviewer #1:**

The aim of this study was to predict clinically significant PCa (CSPCa) by combining the maximal standardized uptake value (SUVmax) based on 68 Ga‑PSMA PET/CT and clinical indicators in men with gray zone PSA levels. The topic of the study is useful for clinician to selected a optimal imaging techniques. However, there are some weakness in this study.

- **Reply:** We thank the Reviewer for succinctly summarizing the highlights of our study. We have revised the original paper based upon your suggestions and marked all changes by using the "Track Changes" function of the word processing program.

1. How many radiologists evaluated the PI-RADS? Was there a consistency assessment?

- **Reply:** We would like to thank the reviewer for the constructive comments. In our study, two radiologists evaluated the PI-RADS score and two nuclear medicine physicians evaluated the ^68^Ga-PSMA PET/CT results independently. In addition, we have analyzed the consistency assessment in PI-RADS score and ^68^Ga-PSMA PET/CT results. We have added these instructions in the "Materials and methods" and "Results" sections per your recommendation. Please see Page 9, Line 13-14 and Page 10, Line 6-8.

2. Did all patients confirmed by pathology? If the result of biopsy were benign disease (such as BPH or acute or chronic prostatitis), how to avoid the error of biopsy?

- **Reply:** We thank the reviewer for the opportunity to clarify this point. All patients were confirmed by pathology. If the results of biopsy were benign disease, patients would undergo a minimum of 1-year follow-up through PSA, prostate mpMRI. We have added the statement in the revised manuscript (Page 7, Line 7-9), which is as follows: "Patients for whom the biopsy results were negative have undergone a minimum of 1-year follow-up of PSA and mpMRI"

3. What was the optimal threshold for Suvmax in distinguishing CSPCa and non- CSPCa?

- **Reply:** In our study, the optimal threshold for SUVmax in distinguishing CSPCa and non- CSPCa is 5.15 and we could get the 93.10% of sensitivity and 73.10% of specificity for diagnosing CSPCa. We have added this statement in the revised manuscript. Please see Page 11, Line 20-21 and Page 12, Line 1.

4. Please state the diagnostic criteria that define positive and negative results in 68Ga PSMA PET.

- **Reply:** Thanks for your suggestion. We have stated the diagnostic criteria in the "Materials and methods" section per your recommendation. Please see Page 8, Line 12-18.

5. Table 3, the OR=17492.04 and the P=0.000 were not match the statistical description，please check with professional statisticians.

- **Reply:** We are grateful to the reviewer for an important suggestion. We have re-done the univariate analysis after converting PSAD into categorical variable from a continuous variable. We have changed the results in the revised manuscript. Please see Table 3 and Page 10, Line 17.

6. Can a better model be established based on PSA+PI-RADS+PSMA PET?

-**Reply:** We would like to thank the reviewer for the constructive comments. We have ever established a model based on the SUVmax, PI-RADS score and clinical features. But it didn’t show a better diagnostic performance than the model based on SUVmax and clinical features. As shown in the following figure, Model 1 is established based on SUVmax and clinical features, and Model 2 is established based on SUVmax, PI-RADS score and clinical features. ROC analysis showed that they have the same AUC of 0.927.

7. Please provide imaging pictures of at least two typical cases, including PET/CT and MRI.

- **Reply:** We would like to thank the reviewer for the constructive comments. Following the reviewer's suggestion, we have added two typical case pictures in the revised manuscript. Please see Figure 2, Figure 3; Page 12, Line 7-8, Page 23, Line 8-22 and Page 24, Line 1-7.

**Reviewer #2:**

This retrospective study included patients with gray zone PSA levels and performed a multivariate analysis to predict clinically significant PCa (CSPCa) by combining the maximal standardized uptake value (SUVmax) based on 68Ga-PSMA PET/CT and clinical indicators. The diagnostic sensitivity and specificity of the prediction model were 86.21% and 86.54%, respectively. The hypothesis, objectives, methods and results are correct.

- **Reply:** We greatly appreciate the comment.

Minor comments:

Abstract, Page 3, Lines 3-5: revise phrase (English style).

- **Reply:** We would like to thank the reviewer for the constructive comments. Following the reviewer's suggestion, we have revised the phrase. Please see Page 3, Line 3-5.

Discussion: The Discussion addresses all relevant issues. I suggest stressing the following considerations:

"Prostate cancer requires a multidisciplinary approach, in which many specialties play a key role (Ref: Brausi M, et al). In challeging tumours, such as prostate cancer, there is the need for new powerful biomarkers to be able to improve patient management. Combining imaging, analytical and clinical parameters can provide new biomarkers (ref. Delgado Bolton et al. CNM 2023 AND ref. Delgado Bolton et al. CNM 2021).

- **Reply:** We are grateful to the Reviewer for an important suggestion. We have added these instructions and data in the "Discussion" section per your recommendation. Please see Page 12, Line 18-19, Page 14, Line 22 and Page 15, Line 1-3.
